# The RyR2-R2474S Mutation Sensitizes Cardiomyocytes and Hearts to Catecholaminergic Stress-Induced Oxidation of the Mitochondrial Glutathione Pool

**DOI:** 10.3389/fphys.2021.777770

**Published:** 2021-12-09

**Authors:** Jörg W. Wegener, Ahmed Wagdi, Eva Wagner, Dörthe M. Katschinski, Gerd Hasenfuss, Tobias Bruegmann, Stephan E. Lehnart

**Affiliations:** ^1^Department of Cardiology and Pulmonology, Heart Research Center Göttingen, University Medical Center Göttingen, Georg August University of Göttingen, Göttingen, Germany; ^2^Cluster of Excellence “Multiscale Bioimaging: From Molecular Machines to Networks of Excitable Cells” (MBExC), Georg-August University of Göttingen, Göttingen, Germany; ^3^DZHK (German Centre for Cardiovascular Research), Partner Site Göttingen, Göttingen, Germany; ^4^Institute of Cardiovascular Physiology, University Medical Center Göttingen, Georg August University of Göttingen, Göttingen, Germany

**Keywords:** ryanodine receptor, mitochondria, dantrolene, glutathione redox potential, RyR2 Ca^2+^ leak, mitochondrial oxidation, reactive oxygen species (ROS)

## Abstract

Missense mutations in the cardiac ryanodine receptor type 2 (RyR2) characteristically cause catecholaminergic arrhythmias. Reminiscent of the phenotype in patients, RyR2-R2474S knockin mice develop exercise-induced ventricular tachyarrhythmias. In cardiomyocytes, increased mitochondrial matrix Ca^2+^ uptake was recently linked to non-linearly enhanced ATP synthesis with important implications for cardiac redox metabolism. We hypothesize that catecholaminergic stimulation and contractile activity amplify mitochondrial oxidation pathologically in RyR2-R2474S cardiomyocytes. To investigate this question, we generated double transgenic RyR2-R2474S mice expressing a mitochondria-restricted fluorescent biosensor to monitor the glutathione redox potential (*E*_GSH_). Electrical field pacing-evoked RyR2-WT and RyR2-R2474S cardiomyocyte contractions resulted in a small but significant baseline *E*_GSH_ increase. Importantly, β-adrenergic stimulation resulted in excessive *E*_GSH_ oxidization of the mitochondrial matrix in RyR2-R2474S cardiomyocytes compared to baseline and RyR2-WT control. Physiologically β-adrenergic stimulation significantly increased mitochondrial *E*_GSH_ further in intact beating RyR2-R2474S but not in RyR2-WT control Langendorff perfused hearts. Finally, this catecholaminergic *E*_GSH_ increase was significantly attenuated following treatment with the RyR2 channel blocker dantrolene. Together, catecholaminergic stimulation and increased diastolic Ca^2+^ leak induce a strong, but dantrolene-inhibited mitochondrial *E*_GSH_ oxidization in RyR2-R2474S cardiomyocytes.

## Introduction

In the heart, where ATP consumption is highest of any tissue, Ca^2+^ homeostasis in the mitochondrial matrix and ATP synthesis in the inner mitochondrial membrane are tightly controlled and interacting in a tissue (muscle)-specific manner regulated by the mitochondrial membrane potential (Wescott et al., 2019; Boyman et al., 2020; Boyman et al., 2021). Pathologically altered intracellular Ca^2+^ signaling and impaired bioenergetics due to mitochondrial dysfunction contribute to both ventricular tachyarrhythmia (VT) and heart failure development, at least in part through increased production of reactive oxygen species (ROS) (Zhou and Tian, 2018; Santulli et al., 2021). Under physiological conditions at rest, mitochondrial oxidative phosphorylation and ATP synthesis are accompanied by a low level of cardiac ROS generation mainly through byproducts of the electron transport chain (Murphy, 2009). However, increased stress and enhanced cardiac performance strongly augment mitochondrial respiratory activity significantly increasing ROS production (Bertero and Maack, 2018). Although several enzymatic systems protect cardiomyocyte function through ROS neutralization (Munzel et al., 2017), chronically compromised mitochondrial bioenergetics in heart disease may shift the balance toward increased oxidation of the glutathione (GSH) redox potential (*E*_GSH_) (Bertero and Maack, 2018), especially since an increased energy demand during sustained β-adrenergic stimulation was proposed to weaken mitochondrial antioxidant defense (Bovo et al., 2015). Notably, increased mitochondrial *E*_GSH_ oxidation itself is a quantitative live-cell indicator of a strongly increased ROS production, and hence may identify an early key disease process and mark the beginning of metabolic remodeling in the failing heart (Doenst et al., 2013).

Under physiological conditions of excitation-contraction (E-C) coupling, Ca^2+^ influx through voltage-gated L-type Ca^2+^ channels (Ca_V_1.2) activates mass intracellular Ca^2+^ release via ryanodine receptor type 2 (RyR2) large-conductance channels in the sarcoplasmic reticulum (SR). This Ca^2+^-induced Ca^2+^ release increases the intracellular Ca^2+^ concentration throughout the myofilaments activating myocardial contraction (Bers, 2002), while it accounts for up to two-thirds of cardiac ATP consumption (Suga, 1990; Meyer et al., 1998). Vice versa, for cardiac relaxation intracellular Ca^2+^ is decreased mainly via the SR Ca^2+^-ATPase (SERCA2a) and extruded by electrogenic trans-sarcolemmal Na^+^/Ca^2+^ exchange (NCX). Hence, diastolic Ca^2+^ cycling accounts for an additional ∼30% of total energy consumption at baseline in healthy cardiomyocytes and in human heart tissue (Gibbs et al., 1988; Meyer et al., 1998).

Interestingly, pathophysiological changes in cytosolic Ca^2+^ cycling are widely accepted as a central cause of arrhythmias and contractile dysfunction in atrial and ventricular tissue (Bers, 2002; Alsina et al., 2019; Brandenburg et al., 2019; Dridi et al., 2020; Berisha et al., 2021; Boyman et al., 2021; Sadredini et al., 2021; Santulli et al., 2021). In this context, increased mitochondrial ROS generation can contribute to altered Ca^2+^ handling and vice versa, which may furthermore constitute a vicious cycle in cardiomyocytes (Zhou and Tian, 2018; Santulli et al., 2021). However, the quantitative and molecular mechanisms if and how acutely increased SR Ca^2+^ leak causes excess mitochondrial Ca^2+^ uptake and ROS generation have been and continue to be controversially discussed (Mallilankaraman et al., 2012; Antony et al., 2016; Kohlhaas et al., 2017; Wescott et al., 2019; Boyman et al., 2020; Rossini and Filadi, 2020; Boyman et al., 2021; Santulli et al., 2021). Nonetheless, the largest known ion channel, RyR2, contains a relatively high number of redox-reactive cysteine residues and hence provides an important molecular substrate for increased ROS generation in cardiomyocytes (Santulli and Marks, 2015). For instance, RyR2 is oxidized in atrial tissue *in vivo* in a genetic model of catecholaminergic polymorphic ventricular tachycardia (CPVT), RyR2-R2474S^+/−^ knockin mice, with increased susceptibility for pacing-induced atrial fibrillation (Shan et al., 2012).

Interestingly, while CPVT has been initially characterized solely as an arrhythmogenic cardiomyopathy (Yano et al., 2009), subsequently seizures originating in the brain (Lehnart et al., 2008; Lieve et al., 2019) and glucose intolerance in the Langerhans cells of the pancreas have been demonstrated in humans and mouse models (Santulli and Marks, 2015; Santulli et al., 2015). Although the majority of biophysically investigated RyR2 missense mutations point to a gain-of-function defect following protein kinase A phosphorylation both at the recombinant and native single-channel level (Wehrens et al., 2003; Lehnart et al., 2004; Tester et al., 2007; Lehnart et al., 2008), the role of PKA-dependent RyR2 phosphorylation remains controversial (Leenhardt et al., 2012; Wleklinski et al., 2020) since, e.g., inhibition of cardiac CaMKII by gene therapeutically applied CaMKII inhibitory peptide, but not PKA inhibitory peptide, effectively suppressed ventricular arrhythmias in a murine and an iPSC-CM model of CPTV (Bezzerides et al., 2019). However, a general consensus exists that adult cardiomyocytes and intact hearts from knockin mice expressing different gain-of-function RyR2 mutations are susceptible for significantly increased diastolic Ca^2+^ leak and Ca^2+^ waves following β-adrenergic stimulation (Lehnart et al., 2008; Fernandez-Velasco et al., 2009; Liu et al., 2009; Uchinoumi et al., 2010; Leenhardt et al., 2012; Borile et al., 2021). Strikingly, consistent with the human phenotype of a normal left-ventricular structure and function at rest, morphological and contractile changes have been excluded in a distinct independent RyR2-R2474S knockin mouse with a clear β-adrenergic driven phenotype of Ca^2+^ triggered cardiac VT (Uchinoumi et al., 2010). Given that the altered SR Ca^2+^ leak/load balance needs to be acutely compensated at the cost of increased ATP consumption during catecholaminergic stimulation (Eisner et al., 2020), CPVT-affected hearts may face a challenging metabolic state during episodes of transient intracellular Ca^2+^ overload and β-adrenergic stimulation, which may lead to increased mitochondrial Ca^2+^ load with excessive ROS generation and contribute to electrical and contractile cardiomyocyte dysfunction (Bers, 2014; Wescott et al., 2019; Boyman et al., 2021).

In the failing heart, ROS signaling has been established as a central process of the organic disease progression (Heymes et al., 2003; Yano et al., 2005; Guo et al., 2007; Akki et al., 2009). Increasing evidence suggests that excessive ROS production can lead to chronic cardiac pathologies, such as apoptosis, fibrosis, and hypertrophy jointly promoting the maladaptive ventricular remodeling (von Harsdorf et al., 1999; Cucoranu et al., 2005; Takimoto et al., 2005; Hori and Nishida, 2009; Santulli et al., 2021). Recently, the mechanisms that temporally and spatially link excess ROS and Ca^2+^ as two principal detrimental factors have been addressed in the context of diabetic cardiomyopathy. Acute elevation of the extracellular glucose concentration induces cardiomyocyte ROS production through O-GlcNAcylation of CaMKIIδ and activation of NOX2 (NADPH oxidase type 2) resulting in increased ROS production in the cytosol but not in the mitochondria (Lu et al., 2020). On the other hand, RyR2-R2474S^+/–^ knockin mice exhibiting catecholamine-induced intracellular Ca^2+^ leak subsequently revealed mitochondrial dysfunction, increased ROS production, increased atrial RyR2 channel oxidation, and arrhythmia susceptibility (Xie et al., 2015). Moreover, inhibitory pharmacological treatment selectively targeting the RyR2 Ca^2+^ leak or genetic inhibition of mitochondrial ROS production both prevented atrial fibrillation, indicating that RyR2 leakage and mitochondrial ROS generation may indeed constitute a self-enforcing subcellular cycle that can set off arrhythmias (Xie et al., 2015). However, the acute mechanism and hence direct but transient link of the catecholamine-stimulated RyR2-R2474S^+/–^ Ca^2+^ leak on mitochondrial ROS production have not been investigated previously.

Novel transgenic reporter mouse lines expressing the Grx1-roGFP GSH redox biosensor in a cardiomyocyte-restricted manner targeted to either the cytoplasm or the mitochondrial matrix have been established previously (Swain et al., 2016). Thus, cardiomyocytes from Grx1-roGFP biosensor mice allow the direct live-cell monitoring of changes in compartmentalized - cytosolic vs. mitochondrial - *E*_GSH_ (Swain et al., 2016), as demonstrated acutely in response to high extracellular glucose concentrations recently (Lu et al., 2020).

The objective of the present study is to investigate mitochondrial and cytosolic *E*_GSH_ changes during pacing-controlled contractions in RyR2-R2474S cardiomyocytes and intact hearts in a knockin mouse model of CPVT. To investigate the interplay between acute catecholamine-stimulated increased SR Ca^2+^ leak and mitochondrial oxidation changes, we crossed the mitochondrial matrix-targeted Grx1-roGFP (mito-Grx1-roGFP) redox biosensor line with mitochondrial redox-competent C57BL/6N background RyR2-R2474S^+/–^ knockin mouse strain by heterozygous breeding (Lehnart et al., 2008; Nickel et al., 2015; Swain et al., 2016). While double transgenic mito-Grx1-roGFP:RyR2-R2474S^+/–^ (RyR2-R2474S dTG) cardiomyocytes showed a significant *E*_GSH_ increase during pacing-evoked contractions following β-adrenergic stimulation compared to RyR2-WT control cardiomyocytes, *ex vivo* perfusion of isoproterenol-stimulated RyR2-R2474S dTG hearts significantly extended and confirmed the cellular findings. Importantly, acute treatment of RyR2-R2474S dTG-cardiomyocytes with the RyR channel blocker dantrolene diminished this *E*_GSH_ increase.

## Materials and Methods

### Double-Transgenic and Single-Transgenic Mouse Lines

All animal procedures were reviewed by the institutional animal care and use committee of the University Medical Center Göttingen and the veterinarian state authority (protocol T11/2; LAVES, Oldenburg, Germany) in line with Directive 2010/63/EU of the European Parliament. Isolated cardiomyocytes and hearts were harvested either from heterozygous RyR2-R2474S dTG or RyR2-WT control mice expressing the mitochondria-restricted *E*_GSH_ biosensor in cardiomyocytes in the C57BL/6N background (Lehnart et al., 2008; Nickel et al., 2015; Swain et al., 2016). RyR2-WT control (mito-Grx1-roGFP2^+/–^:RyR2^+/+^) and RyR2-R2474S dTG (mito-Grx1-roGFP2^+/–^:RyR2^R2474S/+^) mice of either sex were humanely euthanized between 12 and 28 weeks age for heart extraction and/or ventricular cardiomyocyte isolation. In addition, cardiomyocytes isolated from single-transgenic (sTG) mouse hearts expressing the biosensor in the cytosol [cyto-Grx1-roGFP2^+/–^) were investigated following protocols reported previously (Swain et al., 2016)].

### Redox Biosensor Measurements and *E*_GSH_ Calculation in Isolated Cardiomyocytes

Ventricular cardiomyocytes were isolated and quality controlled as described previously (Wagner et al., 2014; Peper et al., 2021). Isolated cardiomyocyte suspensions were plated onto laminin-coated glass coverslips (Imaging Dish CG 1.0, Zellkontakt, Nörten-Hardenberg, Germany) at room temperature. Only quiescent brick-shaped cardiomyocytes with cross-striations visualized at high contrast (e.g. see brightfield image below) were used if they contracted regularly in capture to electrical field stimulation (EFS; 4 ms, ± 20 V biphasic, 0.5 Hz, Myopacer, IonOptix, Westwood, MA, United States). Pacing-responsive cardiomyocytes then underwent repeated EFS-trains for 10, 30, and 60 s at 0.1, 1, or 3 Hz, respectively, for up to 20 min (see Supplementary Figure 1). Isolated cardiomyocytes developing sustained spontaneous contractile activity with a frequency above >0.1 Hz after EFS-pacing or during live-cell imaging were excluded from further analysis.

Cardiomyocyte redox biosensor measurements were performed on an inverted epifluorescence microscope (Axiovert Observer, Zeiss, Oberkochen, Germany) equipped with a 40x oil objective (Fluar 40x/1.3, Zeiss, Oberkochen, Germany). The roGFP2 biosensor was excited at 405 and 488 nm using a monochromator with 5 nm bandwidth (Polychrome V, Till Photonics, Gräfelfing, Germany). The emitted light was detected above >530 nm (filter set #49002, Chroma, Olching Germany) and recorded with a CMOS camera (PrimE, Photometrics, Birmingham, United Kingdom). Biosensor signal changes are represented as intensity ratio obtained at 405/488 nm excitation. Images were acquired at 1 Hz and single-cell ROIs were analyzed offline after background subtraction using proprietary software (Visiview version 3.1, Visitron Systems, Puchheim, Germany).

Cardiomyocytes with baseline intensity ratios below 0.15 were paced in series at 0.1, 1, or 3 Hz, respectively, for 10, 30, and 60 s while the redox biosensor signal changes were continuously monitored (for a schematic of the experimental protocol, please refer to Supplementary Figure 1). The non-paced interval between each pacing train lasted 2–3 min to establish steady-state conditions. Only after the baseline redox biosensor signal has reached a stable baseline steady-state, the subsequent EFS pacing period was executed. H_2_O_2_ (200 μM) was applied at the end of each experiment to confirm the maximal exogenous increase in intensity ratio (positive control) to capture the maximally oxidized state of the redox biosensor. In some experiments, dithiothreitol (DTT, 1 mM) was applied additionally after H_2_O_2_ treatment to obtain the maximal decrease of the redox roGFP2 biosensor signal for calculation of the glutathione redox potential (*E*_GSH_). Isoproterenol (100 nM) and dantrolene (5 μM) were applied acutely for 10 min or incubated for 120 min as indicated, respectively, before the start of the biosensor imaging experiment. All experiments were performed in the HEPES-buffered bath solution (composition in mM: NaCl 140, KCl 5.4, MgCl_2_ 1.2, CaCl_2_ 1.2, Na_2_HPO_4_ 0.3, HEPES 10, glucose 10; pH adjusted to 7.4 with NaOH).

*E*_GSH_ (glutathione redox potential) calibration was performed by protocols reported previously (Gutscher et al., 2008; Swain et al., 2016). *E*_GSH_ values were calculated by analysis of the fluorescence intensities at 405 and 488 nm after treatment with H_2_O_2_ (maximal oxidation) and DTT (maximal reduction). The redox potential E^0^′ for roGFP2 was estimated to −280 mV according to protocols published previously (Dooley et al., 2004).

### Whole Heart Imaging

Hearts from mito-Grx1-roGFP2^+/–^:RyR2^+/+^ mice (WT control) and mito-Grx1-roGFP2^+/–^:RyR2^R2474S/+^ mice (dTG) were quickly extracted after cervical dislocation and immediately placed in ice-cold Dulbecco’s phosphate-buffered solution. Non-cardiac tissue was discarded, and the heart perfused via the aorta with Tyrode’s solution (containing in mM: 1.8 CaCl_2_, 140 NaCl, 5.4 KCl, 2 MgCl_2_, 10 Glucose, 10 HEPES; pH adjusted to 7.4 using NaOH, bubbled with 100% O_2_ at 37°C). A bipolar epicardial electrogram was obtained using a silver electrode in contact with the right atrium and a metal spoon at the heart’s apex. The signal was amplified (Animal Bio Amp FE136), recorded using PowerLab 16/35, and analyzed with LabChart 8.1.18 software, all ADinstruments, Oxford, United Kingdom. Hearts were placed in the optical path of a macroscope (MVX10, MVLPAPO1x, NA: 0.25, Olympus, Hamburg, Germany) connected through a light-guide (Ø 2 mm, NA 0.5) with a LEDHub (Omicron-Laser, Rodgau, Germany). Hearts from WT control and dTG mice showed spontaneous beating rates of 286 ± 30 (*n* = 4) and 217 ± 32 (*n* = 3) bpm under untreated conditions, respectively, which increased to 382 ± 38 (*n* = 4) and 322 ± 50 (*n* = 3) bpm after β-adrenergic stimulation, respectively.

Hearts expressing the roGFP2 sensor were excited using the 385 nm LED and 460 nm LED within the LEDHub and the bandpass filters 385/26 and 475/23 nm (AHF, Tübingen, Germany), respectively. The light was sent to the epicardial surface and the emitted light was captured by the camera using the filter set F46-002XL (AHF, Tübingen, Germany) without the excitation filter. Ratiometric images were acquired at a frame rate of 0.1 Hz using a custom-made software to synchronize both LEDs with the Zyla camera (Andor, Oxford Instruments, Oxford, United Kingdom) through NI 9263 CompactDAQ (National Instruments, Austin, TX, United States). Imaging and cardiac electrogram recording were performed during stable sinus rhythm and after isoproterenol stimulation (33 nM). At the end of each experiment, H_2_O_2_ (200 μM) was applied followed by DTT (4 mM) to calibrate and calculate *E*_GSH_.

### Confocal Imaging of Cardiomyocytes

Cardiomyocyte endomembrane structures were stained using the lipophilic fluorescent dye di-8-ANEPPS (50 μM, Molecular Probes, Eugene, OR, United States) for 15 min at room temperature followed by two washing steps with buffer solution as reported previously (Wagner et al., 2014). Images were acquired with a confocal laser scanning microscope (LSM710, Carl Zeiss, Oberkochen, Germany) with a Plan-Apochromat × 63/1.4 oil Ph3 objective using 16x line averaging. For combined imaging of roGFP2 expressed in the mitochondrial matrix, the fluorescence excited at 490 nm was detected between 500 and 550 nm, and for di-8-ANEPPS, the fluorescence excited at 480 nm was detected between 550 and 650 nm. Pinhole was set to 0.8 AU.

### Chemicals

All chemicals were obtained from Sigma-Aldrich (Merck KgaA, Germany) unless otherwise indicated.

### Data Analysis

Data are reported as intensity ratio (I405/I488 in isolated cardiomyocytes and I385/I475 in whole hearts) or as ratio normalized to the baseline obtained at the start of each recording. Data analysis was performed with Prism 7 (GraphPad Software, San Diego, CA, United States). All data sets have passed a normality test (Kolmogorov-Smirnov). Statistical analysis between RyR2-WT control vs. RyR2-R2474S dTG cardiomyocytes was performed using paired or unpaired ANOVA with Dunnett’s or Tukey’s post-test as appropriate.

## Results

### Baseline Mitochondrial Oxidation at Higher Beating Rates in sTG-Cardiomyocytes

**FIGURE 1:**
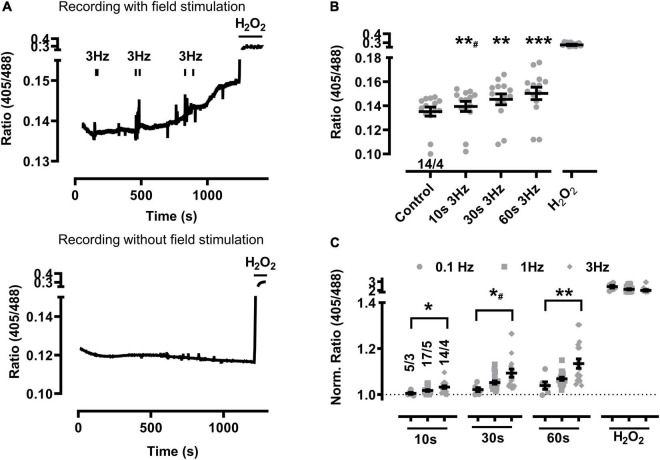
Baseline mitochondrial oxidation at higher beating rates in sTG-cardiomyocytes. **(A)** Representative time course of the redox biosensor I405/I488 nm ratio following repeated EFS-evoked 3 Hz beating trains (*top*) compared to the absence of EFS-pacing in WT control cardiomyocytes (*bottom*). Black bars indicate the start and end of each beating interval lasting 10, 30, and 60 s as summarized by the graphical protocol in Supplementary Figure 1. H_2_O_2_ (200 μM) was added at the end of each experiment to obtain the maximal level of mitochondrial oxidation. Of note, vertical “noise” may originate from aberrant cardiomyocyte contractions. **(B)** Dot plot showing the I405/I488 nm ratio of individual cells and mean values ± SEM. Measurements were obtained under baseline conditions and following EFS-pacing at 3 Hz for the indicated beating durations. Cell/heart numbers are indicated. Paired one-way ANOVA with Dunnett’s post-test. **^#^*P* = 0.006; **P = 0.002; ***P < 0.001. **(C)** Dot plot summarizing individual and mean ± SEM 405/488 nm normalized ratio values recorded at the indicated beating rates and train durations. Ratio values were normalized to baseline at the start of each experiment. Cell/heart numbers are indicated. Unpaired one-way ANOVA with Tukey’s post-test. *P = 0.01; *^#^*P* = 0.02; **P = 0.007.

**FIGURE 2:**
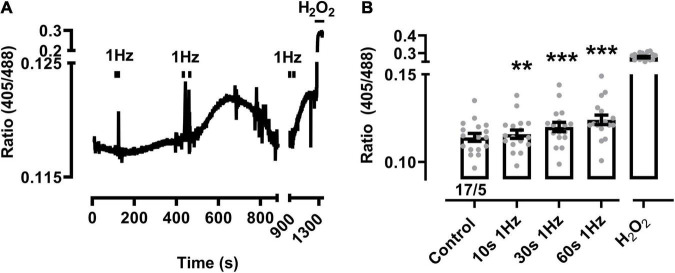
Mitochondrial oxidation following prolonged 1 Hz beating in sTG-cardiomyocytes. **(A)** Representative time course of the redox biosensor I405/I488 nm ratio with pacing-evoked 1 Hz beating trains in a mito-Grx1-roGFP2 cardiomyocyte. Black bars indicate the start and end of each beating interval lasting 10, 30, and 60 s, respectively. H_2_O_2_ (200 μM) was added at the end of the experiment as indicated to confirm the maximal level of mitochondrial oxidation. The interval between 900 and 1,300 s was condensed to show the reversible signal changes after 30 s pacing. **(B)** Dot-bar plot showing the I405/I488 nm ratio of the individual cardiomyocytes and mean values ± SEM. Measurements were obtained under baseline conditions and following EFS-pacing at 1 Hz for the indicated beating durations. Cell/heart numbers are indicated. Paired one-way ANOVA with Dunnett’s post-test. **P = 0.007; ***P < 0.001.

Changes of the biosensor signal were initially characterized at increasing durations of pacing-evoked contractions in cardiomyocytes from sTG-mice expressing the redox reporter Grx1-roGFP2 targeted to either the mitochondrial matrix or the cytosol to confirm the imaging workflow as reported previously (Swain et al., 2016; Lu et al., 2020). Mito-Grx1-roGFP2 cardiomyocytes exhibited an apparent increase of the redox I405/I488 ratio after pacing when compared to non-paced cells which was further increased by elongating the 3 Hz EFS-trains (Figure 1A). On average, 10 s of 3 Hz pacing with optically confirmed contractile pacing-capture was already sufficient to significantly increase mitochondrial oxidization (Figure 1B). Moreover, mitochondrial oxidation was further significantly increased by longer pacing durations both 30 and 60 s, while H_2_O_2_ (200 μM) treatment confirmed the maximal level of the biosensor response (Figure 1B). Likewise, 10 s of 1 Hz pacing considerably increased mitochondrial oxidation which was further enhanced by longer pacing intervals of 30 and 60 s (Figures 2A,B). Of note, the original recording shows that the increase in the redox I405/I488 ratio after 30 s pacing at 1 Hz returned slowly to baseline over several minutes time indicating that the effect of pacing on the biosensor signal is reversible (Figure 2A). In summary, mitochondrial oxidation was significantly increased by pacing at 3 Hz compared to 0.1 Hz beating trains for 10, 30, and 60 s (Figure 1C).

**FIGURE 3:**
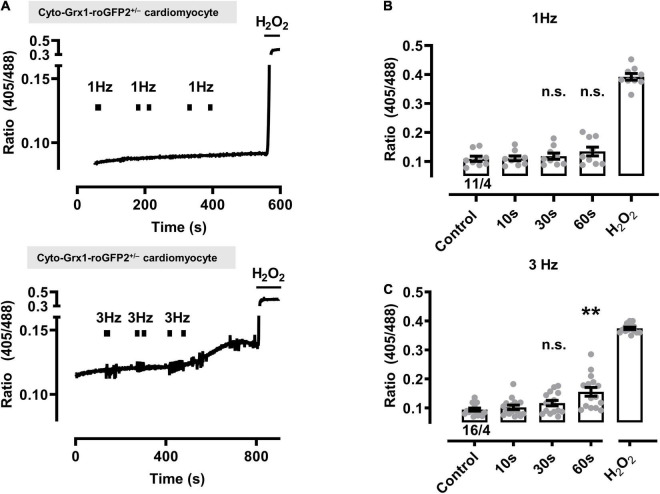
Effect of contractile activity induced by electrical field stimulation (EFS) on the redox sensor signal in cardiomyocytes from cytosolic Grx1-roGFP2^+/–^ mice. **(A)** Representative time course of the redox signal (ratio I405/I488) with contractile activity by EFS at 1 Hz (above) and 3 Hz (below). Marks indicate the start and stop of the stimulation interval which lasted 10, 30, and 60 s, respectively. H_2_O_2_ (200 μM) was added at the end of each experiment to obtain the maximal oxidized value. Up and down reflections of the signal represent low-frequency (<0.1 Hz) contractions of the myocyte. **(B)** Mean values ± SEM of ratio values obtained in cardiomyocytes under control conditions and after EFS at 1 Hz with a 10, 30, and 60 s duration. The signal values of each cardiomyocyte were obtained under control conditions and after the respective pacing. We used 11 cells from four mice as indicated. Statistical analysis was performed by paired one-way ANOVA with Dunnett’s post-test. n.s., non-significant. **(C)** Mean values ± SEM of ratio values obtained in cardiomyocytes under control conditions and after EFS at 3 Hz with a 10, 30, and 60 s duration. We used 16 cells from four mice as indicated. Statistical analysis was performed by paired one-way ANOVA with Dunnett’s post-test. **P = 0.008; n.s., non-significant.

In contrast, the cytosolic biosensor signals in cyto-Grx1-roGFP2 transgenic cardiomyocytes showed only non-significant smaller oxidation increases at 1 or 3 Hz beating, except when the 3 Hz beating rate lasted 60 s, while H_2_O_2_ confirmed the maximal expected response (Figure 3). Taken together, these data indicate that a stronger increase of the beating rate (e.g., from 0.1 to 1 and 3 Hz) leads to significantly increased oxidization of the mitochondrial GSH pool in a time-dependent manner (e.g., from 10 to 30 and 60 s), whereas a significant cytosolic oxidation increase was only observed after the longest (60 s long) 3 Hz beating rate.

### Preserved Transverse Tubule and Mitochondrial Network Interface in sTG-Cardiomyocytes

**FIGURE 4:**
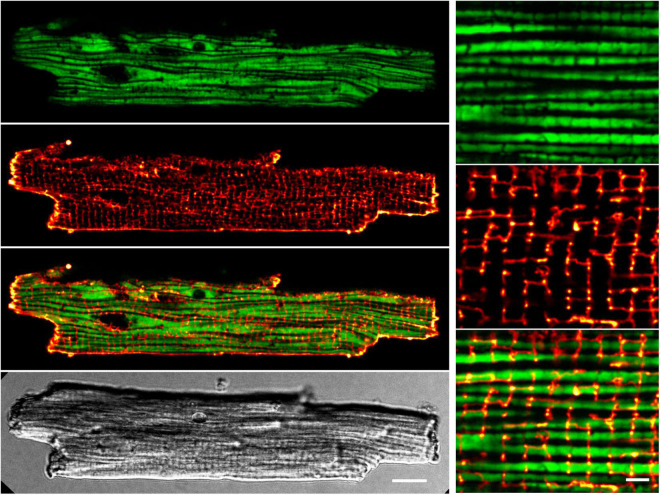
Preserved transverse (T-)tubule and mitochondrial network interfaces in sTG-cardiomyocytes. Confocal live-cell images showing a representative ventricular cardiomyocyte expressing mitochondrial Grx1-roGFP2 after staining with the membrane dye di-8-ANEPPS. *Left* (from top to bottom): (i) roGFP2 auto-fluorescent signals (green LUT) demonstrating the longitudinal chain pattern typical for inter-sarcomeric mitochondria. (ii) di-8-ANEPPS membrane signal (red LUT) confirming a preserved, regular TAT membrane network mainly composed of T-tubule components. (iii) Overlay of the Grx1-roGFP2 and TAT membrane network signals showing a preserved gap-arrangement and confirming network interfaces of T-tubules and mitochondrial membranes. (iv) Cell quality and membrane integrity was confirmed by transmission scanning microscopy with differential interference contrast detecting regular transverse striations and sharp brick-shaped cell edges. Scale bar 10 μm. *Right* (from top to bottom): zoom-in magnifications (i–iii) visualize the distinct mitochondrial and T-tubule compartments and interfaces. Scale bar 2 μm.

Accounting for ∼30% of the cell volume of adult cardiomyocytes, it is anticipated that the regular subcellular inter-sarcomeric mitochondrial distribution is highly ordered between and along the myofilaments, as well as precisely juxtaposed by the junctional SR and transverse tubule (T-tubule) membrane invaginations at both mitochondria poles, where discontinuous T-tubule/SR contacts function as Ca^2+^ release sites containing highly concentrated Ca_*V*_1.2 channels opposed to RyR2 clusters in nanometric proximity (recently reviewed in Rossini and Filadi, 2020). Whereas live-cell confocal mito-roGFP2 imaging showed the expected preserved inter-sarcomeric mitochondrial density apparent as mature longitudinal chain architecture, the transverse-axial-tubule network furthermore revealed the expected invagination network components mainly composed of T-tubules (Figure 4) as reported previously in living mouse cardiomyocytes (Wagner et al., 2012). Apparently, the high T-tubule density regularly intersects the longitudinal mitochondria strands perpendicularly in register (Figure 4 *overlay magnification*). These live-cell data indicate that the transgenic mito-roGFP2 expression did not affect the mechano-sensitive electrical conduction conduits in cardiomyocytes, which is an important prerequisite for a preserved excitation-bioenergetic coupling of the mitochondrial matrix reported recently (Wescott et al., 2019; Boyman et al., 2020; Boyman et al., 2021).

### Increased Mitochondrial Oxidation in RyR2-R2474S dTG-Cardiomyocytes

**FIGURE 5:**
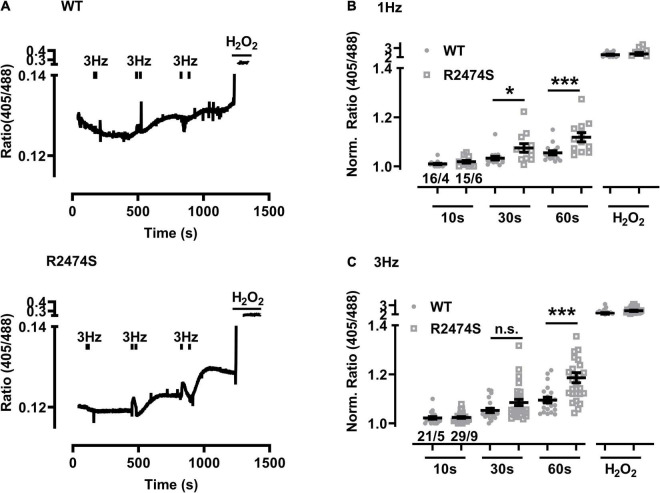
Increased mitochondrial oxidation in RyR2-WT vs. RyR2-R2474S dTG-cardiomyocytes following different beating rates. **(A)** Representative time course of the I405/I488 nm ratio following 3 Hz beating of RyR2-WT control (*top*) and RyR2-R2474S (*bottom*) dTG-cardiomyocytes. Black bars indicate the start and end of each beating interval lasting 10, 30, and 60 s. H_2_O_2_ (200 μM) was added at the end of each experiment to obtain the maximal oxidized value. **(B,C)** Dot plots showing individual and mean ± SEM normalized I405/I488 nm ratio values for the indicated durations either at the 1 Hz **(B)** or 3 Hz **(C)** beating rate. The fluorescent signal values of each cardiomyocyte were obtained under baseline conditions and after the indicated beating trains each lasting 10 s, 30 s or 60 s. Cell/heart numbers are indicated. Unpaired one-way ANOVA with Tukey’s post-test. ***P = 0.001; *P = 0.03; n.s., non-significant.

Next we extended the post-pacing mitochondrial *E*_*GSH*_ imaging to dTG-cardiomyocytes from mouse hearts additionally expressing the heterozygous RyR2-R2474S mutated tetrameric Ca^2+^ release channels vs. RyR2-WT control cells. Apparently, relatively large changes in mitochondrial oxidation were readily evidenced in RyR2-R2474S dTG-cardiomyocytes compared to RyR2-WT control (Figure 5A) particularly following 30 and 60 s of 1 Hz (Figure 5B) and 3 Hz pacing-evoked beating (Figure 5C). Together these data suggest that mitochondrial oxidization is significantly increased in heterozygous RyR2-R2474S dTG-cardiomyocytes by the identical 1 and 3 Hz beating rate pacing-trains compared to RyR2-WT control cells.

### β-Adrenergic Stimulation Exacerbates Mitochondrial Oxidization in RyR2-R2474S dTG-Cardiomyocytes

**FIGURE 6:**
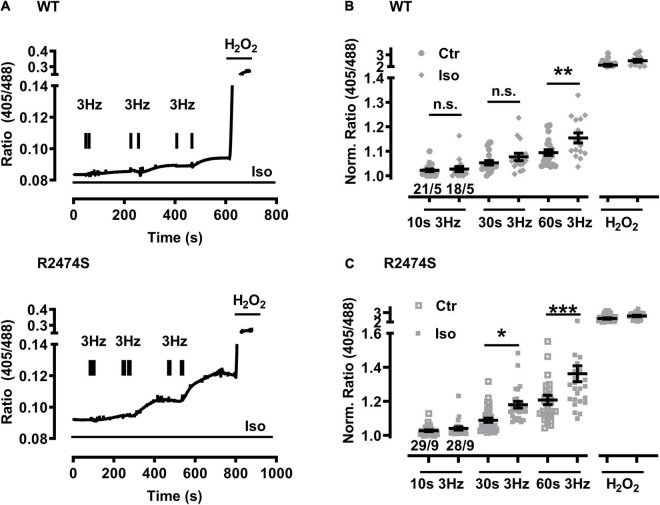
Increased mitochondrial oxidation in WT control and RyR2-R2474S dTG-cardiomyocytes following β-adrenergic stimulation and 3 Hz beating. **(A)** Representative time course of the I405/I488 nm ratio of RyR2-WT control (*top*) and RyR2-R2474S dTG (*bottom*)-cardiomyocytes upon/during isoproterenol (100 nM) stimulation. Black bar pairs indicate the start and stop of the pacing lasting 10, 30, or 60 s. H_2_O_2_ (200 μM) was added at the end of each experiment to obtain the maximal oxidized value. **(B)** Dot blot showing individual and mean values ± SEM of normalized ratio values obtained after EFS at 3 Hz with a 10, 30, and 60 s duration in the absence (open squares) or presence of isoproterenol (100 nM, closed squares) in cardiomyocytes from WT control mice. Numbers indicate the number of cells/number of mice used. Statistical analysis was performed by unpaired one-way ANOVA with Tukey’s post-test. **P < 0.009; n.s., non-significant. **(C)** Dot plot summarizing the individual and mean ± SEM values of the normalized 405/488 nm ratio obtained following 3 Hz beating for 10, 30, and 60 s in the absence (open squares) and presence of isoproterenol (100 nM, closed squares) in RyR2-R2474S dTG-cardiomyocytes. Cell/heart numbers are indicated. Unpaired one-way ANOVA with Tukey’s post-test. ***P < 0.001; *p = 0.03; n.s., non-significant.

**FIGURE 7:**
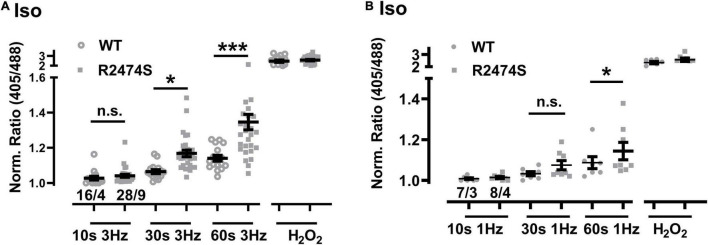
Comparison of the increased mitochondrial oxidation in RyR2-WT control vs. RyR2-R2474S dTG-cardiomyocytes following β-adrenergic stimulation. **(A)** Dot plot showing the individual and mean values ± SEM of the normalized I405/I488 nm ratio values after 3 Hz beating for 10, 30, and 60 s in the presence of isoproterenol (100 nM). Open circles and closed squares reflect the values of the single experiments from RyR2-WT control and Ryr2-R2474S dTG mice, respectively. Cell/heart numbers are indicated. Unpaired one-way ANOVA with Tukey’s post-test. ***P < 0.001; *P = 0.04; **(B)** Dot blot showing individual and mean values ± SEM of normalized ratio values obtained after EFS at 1 Hz with a 10, 30, and 60 s duration in the presence of isoproterenol (100 nM). Open circles and closed squares reflect the values of the single experiments from RyR2-WT control and Ryr2-R2474S dTG mice, respectively. Numbers indicate the number of cells/number of mice used. Statistical analysis was performed by unpaired one-way ANOVA with Tukey’s post-test. *P < 0.02; n.s., non-significant.

**FIGURE 8:**
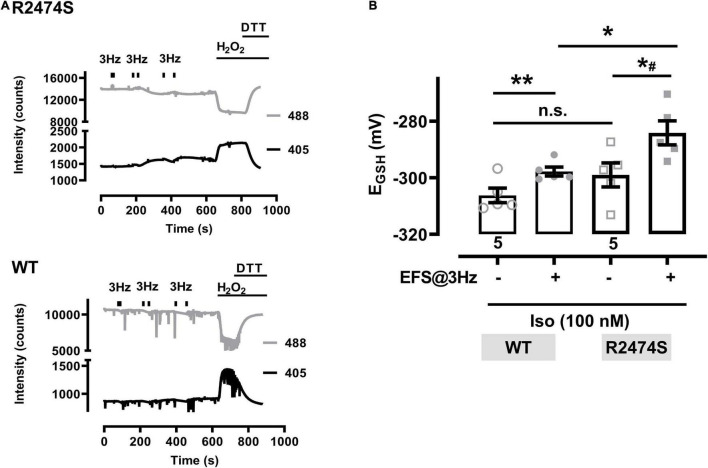
Glutathione redox potentials (E_*GSH*_) in cardiomyocytes from RyR2-WT control and RyR2-R2474S dTG mice. **(A)** Representative trace of the emitted fluorescent intensity signals at 405 and 488 nm excitation in a cardiomyocyte from a RyR2-R2474S dTG mouse (above) and a WT control mouse (below) in the presence of isoproterenol (100 nM). Bars indicate the start and stop of contractile activity induced by EFS at 3 Hz. H_2_O_2_ (200 μM) followed by DTT (1 mM) were applied at the end of the experiment to obtain the maximal and minimal signal values, respectively. **(B)** Calculated *E*_*GSH*_ before and after contractile activity induced by EFS-pacing (3 Hz, 60 s, time point at 600 s) in the presence of isoproterenol (100 nM) in cardiomyocytes from RyR2-WT control and RyR2-R2474S dTG mice. Numbers indicate the number of cells used (five cells from two mice, each). Statistical analysis was performed by a paired (control vs. isoproterenol) or unpaired (RyR2-WT control vs. RyR2-R2474S dTG) Student’s *t*-test. **P = 0.001; *P = 0.02; *^#^*P* = 0.01; n.s., non-significant.

An increased sympathetic tone, exercise, and an increased heart rate provoke the characteristic cardiac CPVT phenotype leading to VT episodes in humans (Lehnart et al., 2004; Danielsen et al., 2018; Baltogiannis et al., 2019). Consequently, isolated cardiomyocytes were exposed to selective β-adrenergic stimulation (isoproterenol 100 nM) and pacing (Figure 6A). First, mitochondrial oxidation was significantly increased by isoproterenol-stimulation in WT control (Figure 6B) and RyR2-R2474S dTG-cardiomyocytes (Figure 6C) when compared to the untreated control cardiomyocytes, respectively. Notably, the isoproterenol-stimulation robustly increased mitochondrial oxidation already at post-30 s beating at 3 Hz in RyR2-R2474S dTG-cardiomyocytes compared to control (Figure 6C). Second, isoproterenol-treatment increased mitochondrial oxidation to a significantly larger extent in RyR2-R2474S dTG-cardiomyocytes compared to RyR2-WT control cells at the 3 Hz beating rate both after 30 and 60 s (Figure 7A) and even already at the lower 1 Hz beating rate after 60 s (Figure 7B). Third, mathematical quantification of the *E*_GSH_ revealed a significant depolarization/oxidation of over 10 mV following isoproterenol-treatment in RyR2-R2474S dTG-cardiomyocytes and a similarly increased oxidation at baseline and isoproterenol-stimulated between RyR2-R2474S dTG-cardiomyocytes and RyR2-WT control cells (Figure 8). Baseline *E*_GSH_ was not different between RyR2-WT control and RyR2-R2474S dTG-cardiomyocytes. Mean calculated *E*_GSH_ values ± SEM (in mV) were −290.9 ± 2.2 (*n* = 8) and −288.8 ± 3.4 (*n* = 9), respectively. As the level of mitochondrial oxidation was overall robustly increased after isoproterenol-stimulation when compared to non-treated control RyR2-R2474S dTG-cardiomyocytes (Figure 7), β-adrenergic stimulation clearly exacerbated the mitochondrial glutathione oxidization.

### Isoproterenol Strongly Increases Mitochondrial Oxidation in Intact RyR2-R2474S dTG-Hearts

**FIGURE 9:**
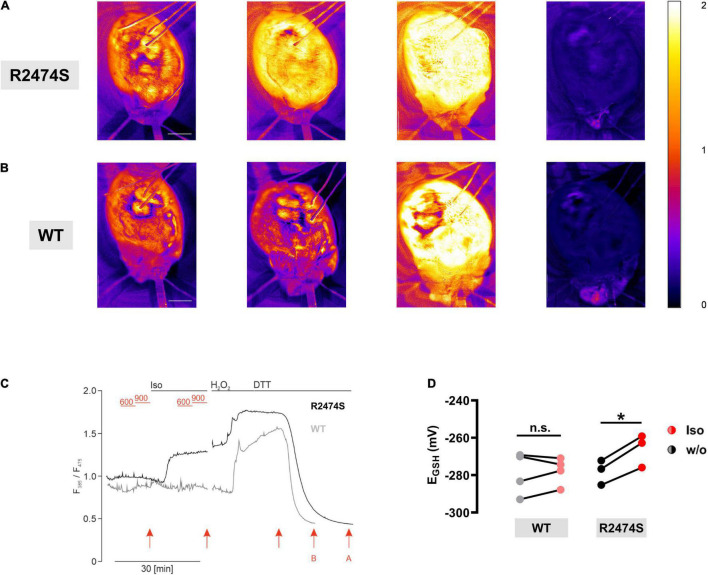
β-adrenergic stimulation induces mitochondrial oxidation in intact RyR2-R2474S hearts. **(A,B)** Color-coded I385/I470 nm ratio comparing a representative RyR2-R2474S dTG-heart **(A)** and a RyR2-WT heart **(B)**. From left to right: (i) baseline control, (ii) following isoproterenol (33 nM) stimulation, (iii) treatment with H_2_O_2_ (200 μM) and iv) DTT (4 mM) (scale bar, 2.5 mm). **(C)** Representative time course of the normalized I385/I475 nm ratio comparing RyR2-WT and RyR2-R2474S dTG-hearts. Black lines indicate each perfusion with 33 nM isoproterenol, 200 μM H_2_O_2_, and 4 mM DTT. The baseline heart rate for RyR2-WT and RyR2-R2474S was 340 bpm and 275 bpm, respectively. In-between, we performed electrical pacing with 600 bpm and 900 bpm (red lines). Red arrows refer to time points for representative images shown in **(A,B)**. **(D)** Paired dot-line plot showing *E*_*GSH*_ calculated before and after application of isoproterenol each for RyR2-WT and RyR2-R2474S dTG-hearts. Each dot represents one heart/experiment. Paired Student’s *t*-test; *P = 0.013; n.s., non-significant.

To monitor mitochondrial oxidation in the intact beating heart, RyR2-WT control and RyR2-R2474S dTG-hearts were Langendorff-perfused and the ventricular left and right epicardial surface imaged with simultaneous surface field-electrogram rhythm recordings. While color-coded mapping of the RyR2-WT dTG-heart apparently captured no major ventricular mitochondrial *E*_GSH_ change following acute β-adrenergic stimulation by isoproterenol (33 nM) in RyR2-WT control hearts (Figure 9B), a robust mitochondrial oxidation increase was evident in the beating RyR2-R2474S dTG-heart (Figure 9A). Meanwhile, both genetic heart groups showed the expected maximally increased oxidation by H_2_O_2_ vs. a maximally decreased oxidation after adding DTT (Figures 9A–C).

Comparing the isoproterenol-stimulated *E*_GSH_ changes confirmed a significant mitochondrial oxidation increase in RyR2-R2474S dTG-hearts, which was, however, not observed in RyR2-WT ventricles (Figure 9D). Together, these results significantly extend and further confirm the findings mainly documented in isolated RyR2-R2474S dTG-cardiomyocytes, underlining a significantly more oxidized mitochondrial state in beating dTG-hearts following β-adrenergic stimulation evidenced by the pronounced changes of the redox signal I385/I475 ratio monitoring acute *E*_GSH_ tissue changes.

### Dantrolene Prevents Mitochondrial Oxidation in RyR2-R2474S dTG-Cardiomyocytes

**FIGURE 10:**
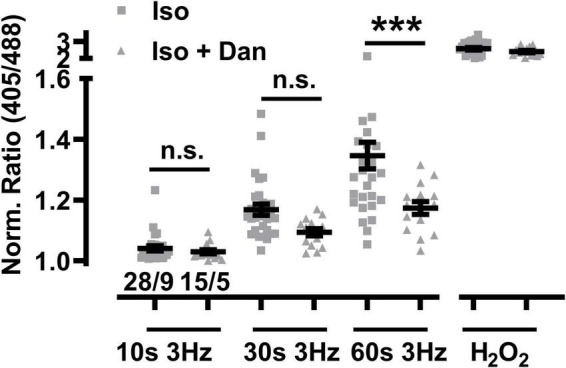
Dantrolene diminishes mitochondrial oxidation in β-adrenergic stimulated RyR2-R2474S dTG cardiomyocytes. Dot plot showing the individual and mean ± SEM values of the normalized I405/I488 nm ratio recorded after 3 Hz beating for 10, 30, and 60 s in the presence of isoproterenol (100 nM) either with or without dantrolene pre-treatment (5 μM for 2 h). Triangles and squares represent individual experiments ± dantrolene as given by the legend. Cell/heart numbers are indicated. Unpaired one-way ANOVA with Tukey’s post-test. ***P < 0.0002; n.s., non-significant.

Dantrolene is used clinically as acute RyR1 channel blocker targeted to skeletal muscles and other tissues as emergency intervention to treat patients who develop life-threatening malignant hyperthermia episodes induced by certain anesthetic drug compounds during surgical interventions. Recent studies demonstrated that dantrolene may also cause beneficial antagonistic effects against the chronic cardiac RyR2 channel Ca^2+^ leak in human diseased cardiomyocytes isolated from failing hearts (Hartmann et al., 2017). Therefore, the acute potential of the large-conductance channel-inhibitory efficacy of dantrolene against mitochondrial oxidation was investigated in RyR2-R2474S dTG-cardiomyocytes following β-adrenergic stimulation (isoproterenol 100 nM). Strikingly, dantrolene pre-treatment (5 μM for 2 h) significantly decreased the large mitochondrial oxidation after 3 Hz 60 s-beating and non-significantly after 3 Hz 30 s-pacing (Figure 10). In summary, these data suggest that a pathologically increased mitochondrial oxidation in isoproterenol-stimulated RyR2-R2474S cardiomyocytes can be partly prevented by pharmacological dantrolene treatment.

## Discussion

This work has examined how mitochondrial *E*_GSH_ oxidation is increased by essential factors in cardiomyocytes with important physiological and pathophysiological consequences that converge at the dynamic interface between RyR2 channel function, sympathetic catecholaminergic stimulation, and contractile activity. Overall, we find that a significant increase following β-adrenergic stimulation significantly changes mitochondrial *E*_*GSH*_ in dTG-cardiomyocytes and hearts, which was further dependent on both the presence of the RyR2-R2474S missense mutation and faster beating rates. Importantly, dantrolene treatment of RyR2-R2474S dTG-cardiomyocytes results in a pronounced reduction of this increased mitochondrial oxidation. We discuss our findings on these interrelated RyR2-R2474S regulatory and *E*_GSH_ driven mitochondrial mechanisms in the context of previously published reports.

Recent quantitative imaging studies established that an increase of mitochondrial Ca^2+^ uptake was necessary to drive inner mitochondrial membrane depolarization and enhanced ATP synthesis in a heart-specific and previously unknown voltage-dependent fashion (Wescott et al., 2019; Boyman et al., 2020; Boyman et al., 2021). This contrasts with heart disease where alterations of mitochondrial *E*_GSH_ may profoundly shift organelle metabolism and thus cardiac bioenergetics (Bertero and Maack, 2018). Beyond bioenergetics, increased mitochondrial *E*_GSH_ oxidation revealed a strongly increased ROS production, which might be of immediate relevance for heart disease progression and metabolic remodeling (Doenst et al., 2013). In analogy, increased mitochondrial oxidation and *E*_*GSH*_ depolarization in RyR2-R2474S cardiomyocytes and hearts indicate a strongly boosted ROS generation during faster beating rates concurrent with β-adrenergic stimulation.

Interestingly, increased SR Ca^2+^ leak and mitochondrial dysfunction have been demonstrated to enhance ROS production in heart disease previously (Zhou and Tian, 2018; Santulli et al., 2021). Remarkably, mitochondrial ROS scavenging attenuated the effects of augmented RyR2 activity leading to proarrhythmic Ca^2+^ waves in Calsequestrin2-null mice serving as a model for CPVT (Hamilton et al., 2020). In line with this view, ROS scavenging by mito-TEMPO was shown to reduce the number of Ca^2+^ waves also in cardiomyocytes from rabbit hearts (Bovo et al., 2012; Cooper et al., 2013). As isoproterenol-stimulated RyR2-R2474S cardiomyocytes exhibit an over threefold increased Ca^2+^ spark frequency that additionally sustains regenerative intracellular Ca^2+^ waves, here the observed robust increase in mitochondrial oxidation will depend both on acute catecholamine stimulation and the beating rate-dependent extent and complex spatiotemporal dynamics of the cell-wide diastolic Ca^2+^ leak reported previously (Lehnart et al., 2008; Xie et al., 2015; Sadredini et al., 2021). However, even under physiological conditions, an increased mitochondrial oxidation was evidenced at higher beating rates in sTG-cardiomyocytes albeit much more pronounced in RyR2-R2474S dTG-cardiomyocytes. For the latter, the faster beating rate clearly led to mitochondrial *E*_GSH_ oxidation and this was most strongly increased after β-adrenergic stimulation.

Tight control of nanomolar diastolic Ca^2+^ levels is essential for normal cardiac function depending on the precise balance between cytosolic Ca^2+^ influx and efflux (Eisner et al., 2020). If the local control mechanisms become compromised, cardiomyocytes will develop arrhythmia and hypercontraction from cytosolic Ca^2+^ overload. This balance is disturbed when the diastolic Ca^2+^ spark frequency is significantly increased, a phenomenon linked molecularly to RyR2 channel gain-of-function defects (Cheng and Lederer, 2008; Bers, 2014). Interestingly, more than a dozen RyR2 mutations originally identified in CPVT patients have been shown to increase Ca^2+^ leak (Jiang et al., 2002; Wehrens et al., 2003; Lehnart et al., 2004; Kontula et al., 2005; Tester et al., 2007; Lehnart et al., 2008; Marjamaa et al., 2011; Shan et al., 2012). However, only two knockin models were confirmed to exhibit exercise-triggered VTs, RyR2-R2474S^+/–^ (Lehnart et al., 2008) and RyR2-R4496C^+/–^ (Priori and Napolitano, 2005; Liu et al., 2006), and are hence reminiscent of the human arrhythmia phenotype diagnosed by exercise stress testing. In line with the *in vivo* pathology, RyR2-R2474S^+/–^ cardiomyocytes exhibited an overall normal diastolic Ca^2+^ spark behavior at rest, but a strong spark frequency increase occurred after isoproterenol treatment (Lehnart et al., 2008). In this context, increased diastolic Ca^2+^ extrusion mainly by SERCA2a and NCX will necessarily consume additional ATP at rest at the cost of concomitantly increased ROS generation (Bers, 2002, 2014). Thus, our data further link increased catecholaminergic RyR2 channel dysfunction in a murine CPVT model to acute mitochondrial oxidation.

In the present study, we monitored the effect of increased contractile activity on *E*_GSH_ in the mitochondrial matrix, while the former process has been long known to correlate with mitochondrial metabolic activity and ROS generation (Gudbjarnason et al., 1962). Since a similar extent of *E*_GSH_ change was excluded in the cytoplasm, NOX2 seems unlikely as ROS source here (Santos et al., 2011). In another context, high extracellular glucose concentrations increased *E*_GSH_ in the cytosol through NOX2 but not in the mitochondrial matrix (Lu et al., 2020). In contrast, we found that mitochondrial oxidization depended on the duration and frequency of dTG-cardiomyocyte beating. This finding is in line with reported changes of NADH in response to a stepwise increase of the beating frequency from 0.1 to 3.3 Hz in guinea-pig cardiomyocytes (Jo et al., 2006). As the increase in the mitochondrial *E*_GSH_ following beating was observed both in RyR2-WT and RyR2-R2474S dTG-cardiomyocytes, a relation with increased SR Ca^2+^ leak seems plausible after pacing-evoked contractile activity reported previously (Satoh et al., 1997). Finally, blocking RyR2 Ca^2+^ leak with dantrolene diminished *E*_GSH_ oxidation as hypothesized previously (Hartmann et al., 2017).

A recent study demonstrated that Ca^2+^ leak increased mitochondrial ROS production in cultured WT rat cardiomyocytes transfected for 48 h with the OMM-HyPer mito-ROS biosensor (Hamilton et al., 2020). An increased ROS production has also been demonstrated during increased beating in healthy cardiomyocytes with the fluorescent probe dichlorodihydrofluorescein, which is confirmed by our sTG-cardiomyocyte data (Heinzel et al., 2006). In rat trabeculae, NADH oxidation was observed at 0.25–2 Hz after 60 s with pronounced ATP hydrolysis (Brandes and Bers, 2002). A similar time frame in response to contractile activity was observed in the present study, where the maximal change of the biosensor signal occurred within 2–3 min after beating. This further agrees with the reported change of the redox signal observed in cardiomyocytes in the same biosensor cardiomyocytes after a high glucose or pH challenge (Lu et al., 2020; Kreitmeier et al., 2021). Since the observed redox changes occurred only after completion of the given beating period, it is suggested to correlate further with the aftercontraction related metabolic activity observed frequently in isoproterenol-stimulated RyR2-R2474S dTG-cardiomyocytes.

Importantly, our report provides the first evidence that *E*_GSH_ changes happen under quasi physiological conditions in the intact beating heart as well. Using the genetically encoded reporter targeted to mitochondria allowed to monitor the average *E*_GSH_ from the whole ventricular, anterior heart surface (Supplementary Videos 1, 2) and to perform a ratiometric imaging excluding any major interference due to cardiac contractions. In comparison to the first description of the glutathione redox sensor mouse lines, we were able to increase the ratio differences from maximal oxidization to reduction to comparable signal-to-noise values to single-cell measurements (see Figures 5, 9). This increase in signal amplitude was the key for the detection of the comparable relatively small steps in oxidization during β-adrenergic stimulation, which we could only detect in the intact hearts of dTG mice expressing the RyR2-R2474S mutated channels. However, in contrast to isolated cardiomyocytes, we could not detect a change in oxidation by additional faster pacing in the spontaneously beating Langendorff hearts. One possible explanation may be that these changes are in comparison subtle and thus still not detectable in this setting. The other is that we focused on the effects of isoproterenol and such changes would occur only after prolonged phases of pacing which we could not implement in these experiments.

In summary, we have shown that contractile activity induced by EFS-pacing in isolated cardiomyocytes increased the mitochondrial *E*_GSH_ to more oxidized levels in a time- and frequency-dependent manner. The increase was most pronounced after β-adrenergic stimulation and in cardiomyocytes expressing the human RyR2-R2474S mutated tetrameric channel. The catecholaminergic *E*_GSH_ increase, however, was significantly attenuated by the pharmacological RyR2 channel antagonist dantrolene. Likewise, we observe a larger increase in the mitochondrial *E*_*GSH*_ toward more oxidized levels in spontaneously beating hearts expressing the RyR2-R2474S mutated channels after β-adrenergic stimulation as compared to control hearts. Hence, we suggest that the RyR2-R2474S mutation induces a metabolic burden in cardiomyocytes through excess ROS generation, especially after β-adrenergic stimulation, tipping the balance from a compensated to a high energetic cost and acutely increased diastolic Ca^2+^ leak, a multifactorial process, which may acutely contribute to the development of the arrhythmogenic CPVT phenotype as proposed for calsequestrin2-null mice previously (Hamilton et al., 2020).

## Data Availability Statement

The original contributions presented in the study are included in the article/Supplementary Material, further inquiries can be directed to the corresponding author/s.

## Ethics Statement

The animal study was reviewed and approved by the Institutional IACUC. This study has not used *in vivo* examination of mice but *ex vivo* investigations, which are not subject to an ethics protocol at our institution.

## Author Contributions

JW and AW performed the research and analyzed the data. JW, TB, DK, and SL wrote the manuscript. DK contributed to the biosensor mouse models and analyzed the data. JW, SL, TB, and GH designed the study. All authors approved the manuscript.

## Conflict of Interest

The authors declare that the research was conducted in the absence of any commercial or financial relationships that could be construed as a potential conflict of interest.

## Publisher’s Note

All claims expressed in this article are solely those of the authors and do not necessarily represent those of their affiliated organizations, or those of the publisher, the editors and the reviewers. Any product that may be evaluated in this article, or claim that may be made by its manufacturer, is not guaranteed or endorsed by the publisher.

## Supplementary Material

Supplementary Video 1 Color coded I385/I475 nm ratio comparing representative RyR2-WT hearts. Showing the ratio images during baseline control, isoproterenol (33 nM) stimulation, treatment with H_2_O_2_ (200 μM), and DTT (4 mM). Scale bar = 5 mm.

Supplementary Video 2 Color coded I385/I475 nm ratio comparing representative RyR2-R2474S dTG-hearts. Showing the ratio images during baseline control, isoproterenol (33 nM) stimulation, treatment with H_2_O_2_ (200 μM), and DTT (4 mM). Scale bar = 5 mm.

## Abbreviations

CPVT: catecholaminergic polymorphic ventricular tachycardia
Dan Dantrolene: dTG double transgenic
DTT: dithiothreitol
EFS: electrical field stimulation
*E*_GSH_: glutathione redox potential
Iso: isoproterenol
roGFP: reduction-oxidation-sensitive green fluorescent protein
ROS: reactive oxygen species
RyR2: ryanodine receptor type 2
SR: sarcoplasmatic reticulum
sTG: single transgenic.
