# Supplementary figures and images for: The RyR2-R2474S Mutation Sensitizes Cardiomyocytes and Hearts to Catecholaminergic Stress-Induced Oxidation of the Mitochondrial Glutathione Pool
Source: Front Physiol. 2021 Dec 9;12:777770. doi: 10.3389/fphys.2021.777770 (PMC8696262; doi:10.3389/fphys.2021.777770)

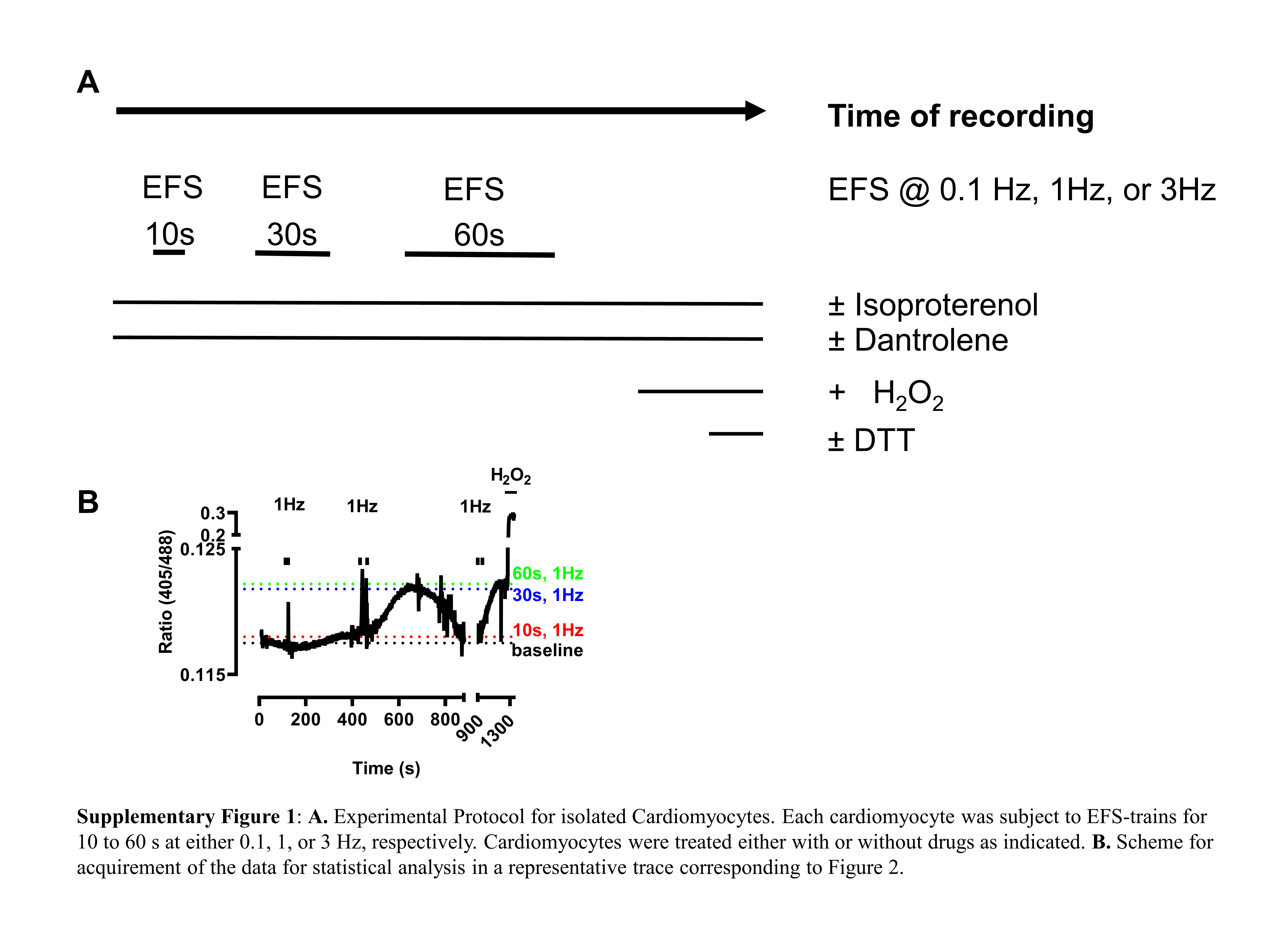

Supplement: Supplementary file 3 [file Image_1.TIF]
